# Cobalt‐Free LiNiO_2_ with a Selenium Coating as a High‐Energy Layered Cathode Material for Lithium‐Ion Batteries

**DOI:** 10.1002/smsc.202300023

**Published:** 2023-06-11

**Authors:** Yuhang Chu, Jinwei Zhou, Wenxin Liu, Fulu Chu, Jinhui Li, Feixiang Wu

**Affiliations:** ^1^ School of Materials Metallurgy and Chemistry Jiangxi University of Science and Technology Ganzhou 341000 China; ^2^ School of Metallurgy and Environment Engineering Research Center of the Ministry of Education for Advanced Battery Materials Hunan Provincial Key Laboratory of Nonferrous Value-added Metallurgy Central South University Changsha 410083 China

**Keywords:** cobalt-free materials, high energy density, LiNiO_2_ cathodes, lithium-ion batteries, selenium coatings

## Abstract

Considering the high cost of cobalt, cobalt‐free lithium nickel oxide (LiNiO_2_), which has an extremely high theoretical specific capacity, has become attractive. However, its application is hampered by continuous irreversible phase changes, unstable interfaces, and particle pulverization. Herein, an ≈35 nm‐thick selenium (Se) coating is applied to modify LiNiO_2_ using a combination of solid‐phase mixing and low‐temperature sintering. After 300 cycles, the selenium‐coated LiNiO_2_ (Se–LNO) cathode exhibits unusually high capacity retention of 82.09% at a charge cutoff voltage of 4.3 V. Furthermore, the coating modification improves the rate performance of the cathode materials, which exhibit considerable specific capacities of 168.7 and 149.6 mAh g^−1^ at current densities of 2 and 5 C, respectively. The significantly enhanced electrochemical performance can be attributed to the ability of the Se coating to enhance the structural stability of the cathode materials by suppressing phase transitions, stabilizing the interface, enhancing the kinetic behavior of the electrode, and reducing particle pulverization. According to the findings of this study, manipulating the Se coating in cathodes may provide a viable path for lower‐cost and higher‐energy‐density Co‐free lithium‐ion batteries.

## Graphical abstract

Se–LNO cathode materials are prepared using a simple melt‐diffusion process at low temperatures, demonstrating the potential for large‐scale use. The Se coating layer minimizes the interfacial side reactions, inhibits phase transitions, and limits particle pulverization, thereby strengthening the interfacial and internal structural stability of the produced cathode materials.

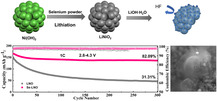

## 1 Introduction

The depletion of fossil fuels and rising pollution issues are pushing the development of high‐specific energy storage components.^[^ ^1^, ^2^ ^]^ Lithium‐ion batteries have been widely investigated and employed as typical energy storage devices because of their high energy density, environmental friendliness, and extended service life. Cathode materials have been extensively studied as the heaviest components of lithium‐ion batteries. Among these, Ni‐based cathode materials such as LiNi_1‐*x*−*y*_Co_x_M_y_O_2_ (M = Mn or Al) are promising candidates because of their high energy density and long cycle life.^[^ ^3^, ^4^, ^5^, ^6^, ^7^, ^8^, ^9^, ^10^ ^]^ Increasing the nickel concentration can enhance the energy density and lower the cost of cathode materials. Furthermore, the use of cobalt, a costly and toxic element, has been minimized in cathode materials. Consequently, studies on producing high‐nickel, low‐cobalt, or even cobalt‐free (Co‐free) Ni‐based cathode materials are attracting considerable attention.^[^ ^11^, ^12^, ^13^ ^]^ Among these cathode materials, Co‐free layered lithium nickel oxide (LiNiO_2_) is undoubtedly the most distinctive, with a maximum theoretical specific capacity of 270 mAh g^−1^ at a higher‐voltage plateau (3.8 V vs Li/Li^+^).^[^ ^14^, ^15^, ^16^, ^17^, ^18^, ^19^, ^20^ ^]^ However, LiNiO_2_ cathodes suffer from deterioration of the material surface and physical structure during cycling due to several reasons. During the synthesis of LiNiO_2_, a large amount of Ni^2+^ cannot be oxidized to Ni^3+^. Because the ionic radius of Ni^2+^ (0.068 nm) is similar to that of Li^+^ (0.076 nm), Ni^2+^ can easily occupy the original position of Li^+^, leading to a phase transition and Li^+^/Ni^2+^ disorder.^[^ ^15^, ^19^ ^]^ Furthermore, Ni^4+^ generated during the charging process reacts with the electrolyte, resulting in the loss of the oxygen lattice and structural collapse of the cathode surface.^[^ ^18^, ^20^ ^]^ Moreover, the tension induced by repetitive volume contraction and expansion during cycling may result in microcracks on the surface of the cathode materials. The electrolyte enters the cathode material via the microcracks and degrades the interior of the cathode material, causing wider fractures and pulverization of the material.^[^ ^21^, ^22^ ^]^

Surface coating is a viable method of addressing these issues and can improve the structural stability of Ni‐rich oxide cathode materials. The coating materials mainly include metal oxides (MgO,^[^ ^9^ ^]^ Al_2_O_3_,^[^ ^23^ ^]^ TiO_2_^[^ ^24^ ^]^ and SiO_2_^[^ ^25^ ^]^ etc.), fluorides (LiF,^[^ ^26^ ^]^ AlF_3_^[^ ^27^ ^]^ and CaF_2_^[^ ^28^ ^]^ etc.), phosphides (AlPO_4_^[^ ^29^ ^]^ and FePO_4_^[^ ^30^ ^]^ etc.), and lithium‐active materials (Li_5_AlO_4_,^[^ ^31^ ^]^ Li_4_SiO_4_,^[^ ^32^ ^]^ Li_2_TiO_3_^[^ ^33^ ^]^ and La_4_NiLiO_8_^[^ ^34^, ^35^ ^]^ etc.). Notably, most coating technologies require considerable energy consumption and lengthy processes, which are not favorable for mass manufacturing. Therefore, the development of a simple and effective coating technique is critical. The use of solid‐phase sintering for coatings is extremely convenient. However, the high‐energy consumption caused by the high melting point of the material to be coated, as well as the inhomogeneity of the coating layer created, restrict the widespread acceptance of this method. Selenium, a monomer with a low melting point (217 °C), melts and flows at lower temperatures.^[^ ^36^ ^]^ As a result, using selenium as a coating material can result in a more uniform coating layer at lower energy. Sun et al. modified the LiNi_0.8_Co_0.1_Mn_0.1_O_2_ cathode material with selenium and discovered that the formation of metal–selenium linkages considerably increased the structural stability, which in turn improved the electrochemical performance.^[^ ^37^ ^]^ Wu et al. improved the cycling and rate performance of the cathode material by applying a sublimation‐induced gas reaction process to selenium‐coated LiNi_0.8_Co_0.1_Mn_0.1_O_2_.^[^ ^38^ ^]^ However, direct selenium modification of Co‐free LiNiO_2_ cathode materials has seldom been documented.

In this study, a facile solid‐phase mixing process combined with low‐temperature sintering was used to prepare a LiNiO_2_ cathode material uniformly coated with selenium. The effects of the selenium coating layer on the crystal structure, morphology, and electrochemical performance of the LiNiO_2_ cathode materials were thoroughly studied. The selenium coating layer can prevent the electrolyte from degrading the active material and limit the occurrence of interfacial side reactions, thus improving the interfacial stability of the cathode material. Furthermore, the coating layer can block the phase transition and prevent particle pulverization, thereby preserving the internal structural integrity of the cathode materials. Based on these, the Co‐free Se‐coated LiNiO_2_ (Se–LNO) cathode materials can exhibit promising electrochemical performance.

## 2 Results and Discussion

**Figure 1:**
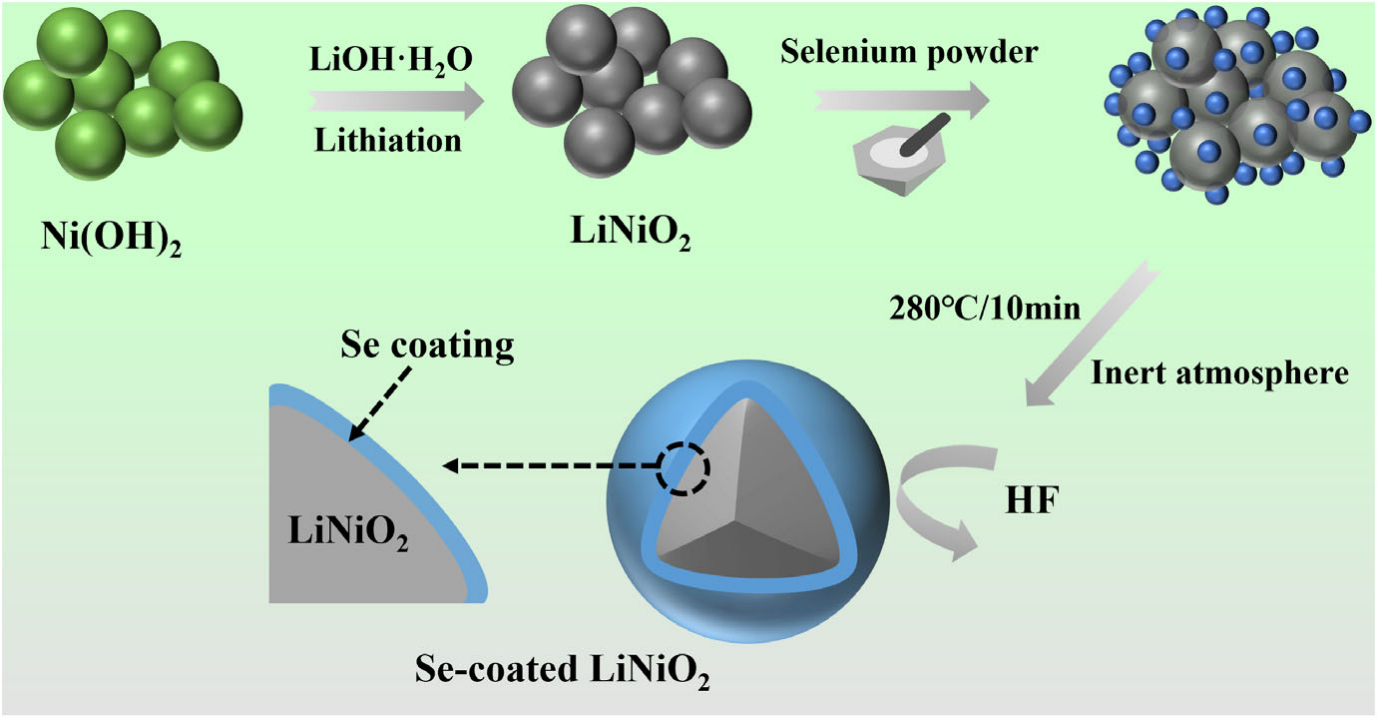
Schematic diagram of the synthesis process for Se–LNO.

**Figure** **1** shows the preparation procedure of Se–LNO. Pure‐phase LiNiO_2_ was coated with Se using a solid‐phase mixing process coupled with low‐temperature sintering. In contrast to the conventional solid‐phase sintering approach, which has a high sintering temperature and produces an inhomogeneous coating layer, this method enables the fast diffusion of molten selenium at a lower temperature to obtain a uniform coating on LiNiO_2_ by taking advantage of the low melting point of monomeric selenium. Furthermore, considering the characteristics of solid‐phase mixing and low‐temperature sintering, this coating process has the advantages of short processing time and low energy consumption.

**Figure 2:**
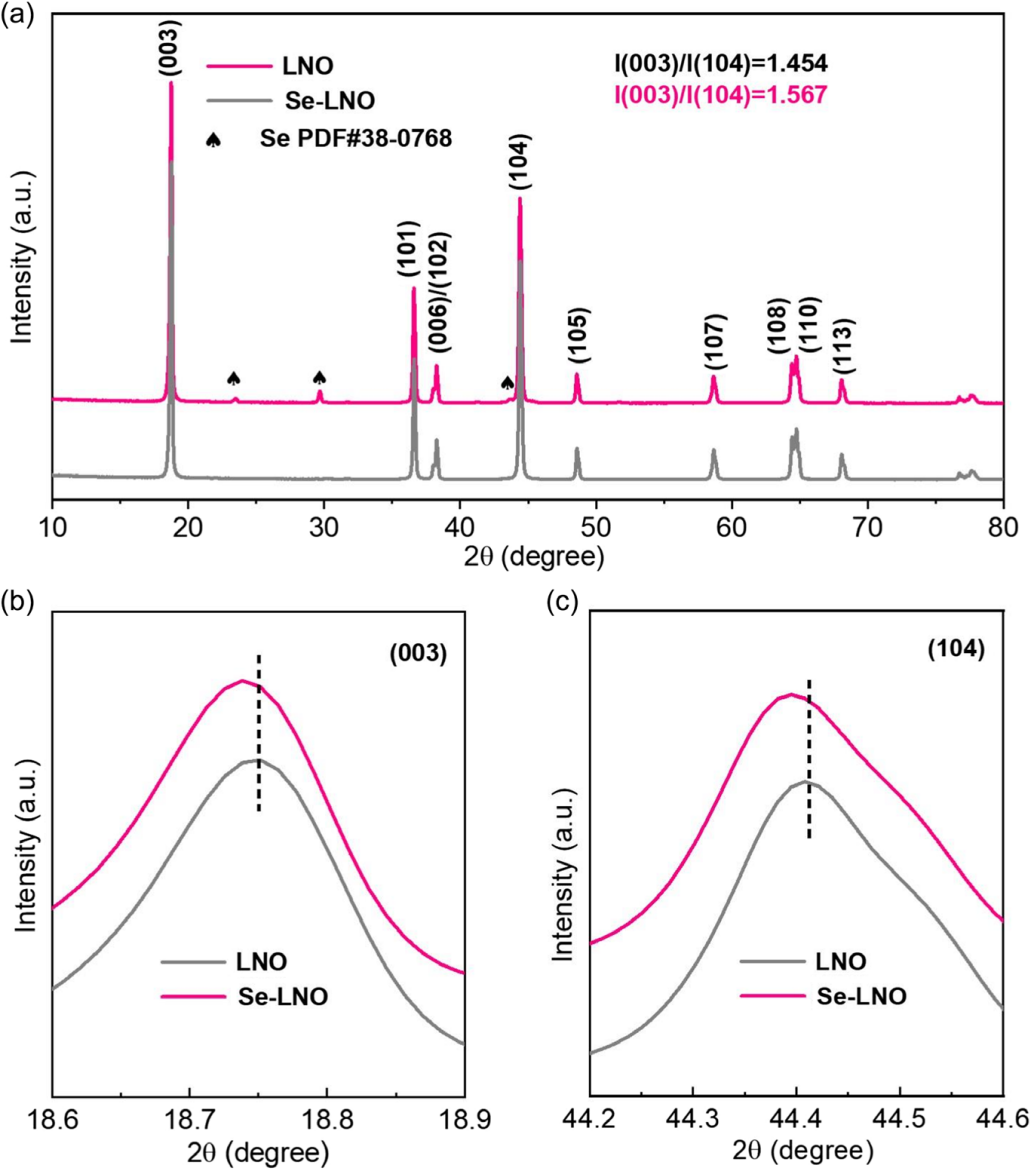
a) XRD results of LNO and Se–LNO and b,c) local amplification of (003) and (104) peaks.

As shown in **Figure** **2** **,** X‐ray diffraction (XRD) was used to characterize the physical phase of the cathode material before and after coating. Figure 2a shows the XRD patterns of LNO and Se–LNO. Both samples exhibited typical *α*‐NaFeO_2_ layered structures in the R‐3 m space group, indicating the successful synthesis of LNO.^[^ ^14^, ^39^ ^]^ Additionally, three low‐intensity peaks of Se (PDF#38‐0768) were observed in the 2*θ* range of 20°–50° in the XRD pattern of Se–LNO, indicating the successful coating of Se onto the surface of LNO. Concurrently, two pairs of split peaks, (006)/(102) and (108)/(110), clearly appeared, indicating that layered structures of the two materials were successfully formed.^[^ ^40^, ^41^ ^]^ The ratio of the (003)/(104) peak strength reflects the degree of Li^+^/Ni^2+^ disorder in the cathode material; the higher the value, the lower the degree of Li^+^/Ni^2+^ disorder.^[^ ^42^, ^43^ ^]^ The *I*_(003)_ */I*_(104)_ ratio of the Se–LNO material was 1.567, which is greater than that of LNO, indicating that the degree of cation mixing decreased after surface modification with Se. In addition, the (003) and (104) peaks of Se–LNO were shifted to smaller angles compared to those of LNO, as can be observed from the local enlargements (Figure 2b,c). This indicates that the c‐axis lattice spacings increase after Se coating, which can improve Li^+^ transport between the layers, thereby improving the dynamics of the charging and discharging processes.

**Figure 3:**
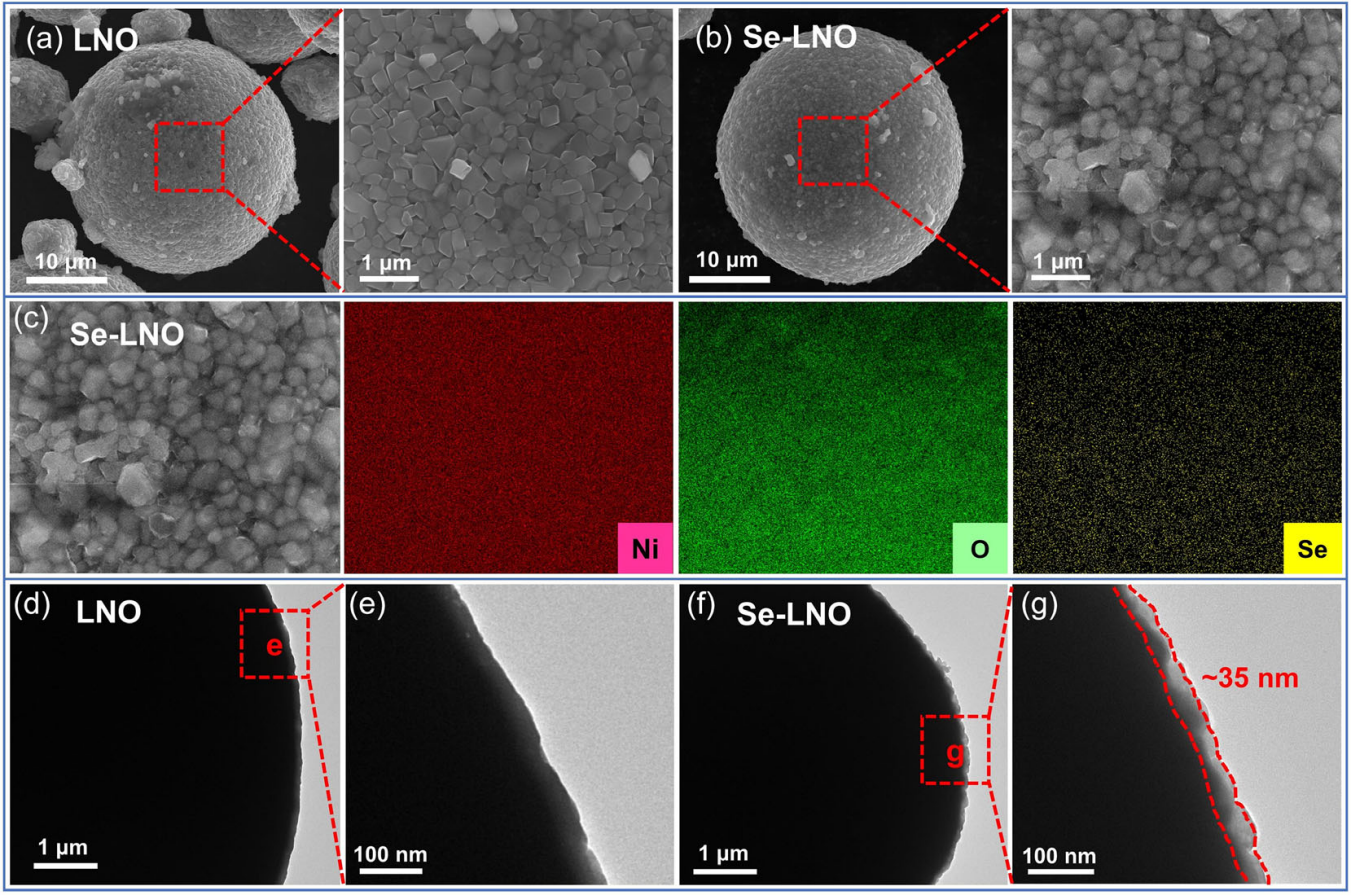
Morphology of the cathode materials before and after coating. a,b) SEM images of LNO (a) and Se–LNO (b); c) mapping of Se–LNO, showing elemental distribution of Ni, O, and Se. d–g) TEM images of LNO (d,e) and Se–LNO (f,g).

Scanning electron microscopy (SEM) and transmission electron microscopy (TEM) were used to observe changes in the surface morphology of the materials. **Figure** **3**a,b shows SEM images of LNO and Se–LNO, respectively. It can be observed that the surface morphology of the cathode material is different before and after the Se coating. Evidently, the surface of the Se–LNO sample exhibited a more uniform and transparent coating layer. Figure 3c shows the elemental mapping of Se–LNO. The uniform distribution of Ni, O, and Se proves that the Se coating layer is homogeneous. The thin and homogeneous Se coating layer was further characterized by TEM. Figure 3d–g shows that the surface of LNO exhibits a smooth single phase, whereas a thin coating layer with a thickness of ≈35 nm can be observed on the Se–LNO surface. Such a uniform and complete coating layer of Se may prevent corrosion of the cathode material by HF and thus stabilize the internal structure of the material, which is expected to enhance the electrochemical performance.

**Figure 4:**
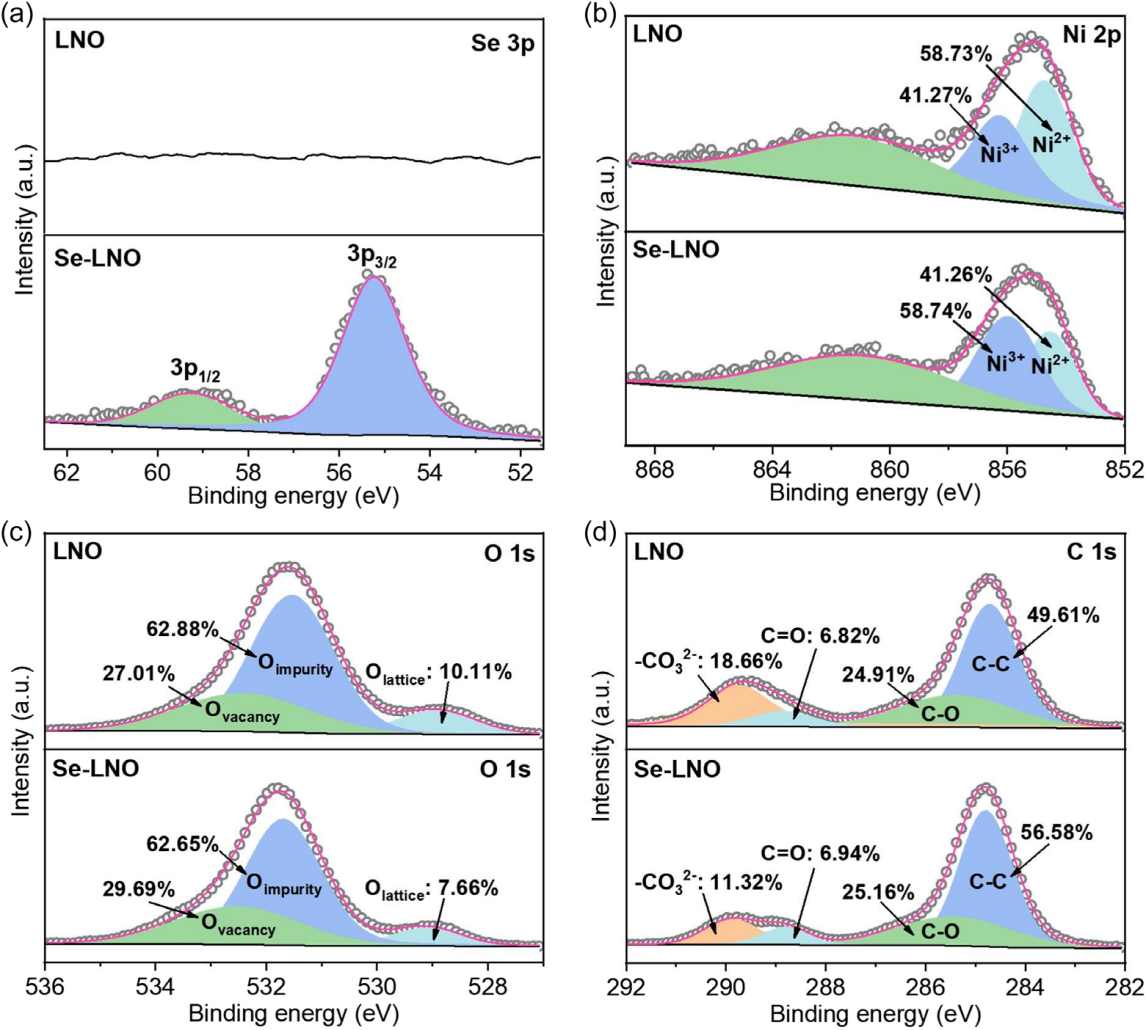
a–d) XPS spectra of LNO and Se–LNO: a) Se 3p, b) Ni 2p, c) O 1s, and d) C 1s.

**Figure** **4** shows the X‐ray photoelectron spectroscopy (XPS) characterization of the cathode materials before and after coating, revealing the surface chemical information of the two samples. Figure 4a shows that the XPS peaks at 55.3 and 59.3 eV corresponding to Se 3p_3/2_ and Se 3p_1/2_, respectively, appear in the Se–LNO sample scans, confirming the presence of Se in Se–LNO.^[^ ^37^ ^]^ As shown in Figure 4b, the binding energy peak of Ni 2p in the Se–LNO sample shifted slightly to the right compared to that of the original sample, indicating that the valence state of the Ni atom was affected by the Se coating. The binding energy of the Ni 2p_2/3_ peak of the coated sample (855.4 eV) was higher than that of the original sample (854.9 eV), indicating the presence of a greater number of Ni^3+^ ions on the surface of Se–LNO. The peaks of Ni^2+^ and Ni^3+^ were separated by the Gaussian fitting method, demonstrating that the original sample had a higher Ni^2+^ content (58.73%) compared to that of the Se–LNO sample (only 41.26%). A higher Ni^2+^ content in layered materials may exacerbate the degree of Li^+^/Ni^2+^ cation mixing.^[^ ^44^ ^]^ This indicates that introducing the Se coating layer can reduce the Li^+^/Ni^2+^ disorder, consistent with the XRD results. The O1s spectrum in Figure 4c includes peaks corresponding to oxygen vacancies (532.5 eV), oxygen impurities (531.7 eV), and lattice oxygen (529.1 eV).^[^ ^45^ ^]^ Se–LNO has a lower percentage of lattice oxygen than LNO at 529.1 eV due to the shielding of the lattice oxygen signal by the Se coating layer. The C 1s spectrum consists of peaks corresponding to CO_3_^2−^ (289.8 eV), C=O (288.8 eV), C—O (285.5 eV), and C—C (284.8 eV).^[^ ^46^ ^]^ The percentage of CO_3_^2−^ indicates that Li_2_CO_3_ content decreases from 18.66% to 11.32% with the addition of Se (Figure 4d), confirming the reduction of Li_2_CO_3_ on the surface of Se–LNO. This observation suggests that surface modification with Se can minimize the residual lithium on the surface of cathode materials, thus stabilizing the surface and interface.

**Figure 5:**
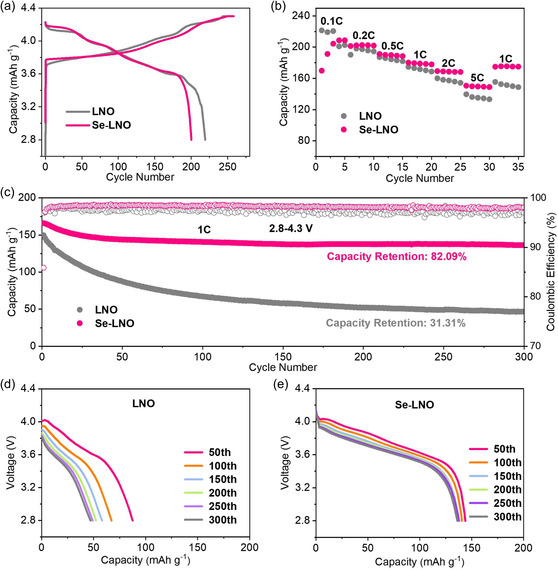
Electrochemical properties of LNO and Se–LNO at cutoff voltages of 2.8–4.3 V. a) Initial charge–discharge curves, b) rate performance, c) long‐term testing at 1 C over 300 cycles and d,e) the changes of charge–discharge curves with cycles.

**Figure** **5**a shows the initial charge and discharge curves of the two samples at 0.1 C (1 C = 180 mAh g^−1^). Compared with LNO, the Se–LNO sample displayed significantly smoother charge/discharge profiles (particularly within the 4.0–4.3 V), indicating that phase transformations in the highly delithiated state are suppressed. This suggests the role of the Se coating layer in suppressing the phase transition at high‐voltage states. In addition, the initial capacity of Se–LNO was 200.1 mAh g^−1^, which is slightly lower than that of LNO (219 mAh g^−1^). This is because Se is not electrochemically active and loses some of its specific capacity when used as a coating. Figure 5b shows the rate performances of the LNO and Se–LNO samples. Notably, the specific capacity of the Se–LNO cathode material exhibited a gradual increase at 0.1 C for five cycles. This is because the Se coating layer is a non‐lithium‐ion conductor, and Se–LNO must be activated by a small current for a few turns before reaching its capacity. After activation at 0.1 C, the specific capacities of Se–LNO were higher than those of the unmodified LNO at 0.2, 0.5, 1, 2, and 5 C. In particular, the Se–LNO sample exhibited average specific capacities of 168.7 and 149.6 mAh g^−1^ at current densities of 2 and 5 C, respectively, which are considerably higher than those of LNO (157.2 and 135.7 mAh g^−1^, respectively). The cycling performances of LNO at a current density of 1 C before and after coating are shown in Figure 5c. Compared with LNO, Se–LNO demonstrated better cycling stability. The specific capacity of the Se–LNO cathode material was 136.3 mAh g^−1^ after 300 cycles, with a capacity retention rate of 82.09%, whereas that of LNO was only 46.9 mAh g^−1^ after 300 cycles, with a capacity retention rate of 31.31%. In addition, Se–LNO showed higher coulombic efficiency than LNO. Compared to LNO, Se–LNO showed a longer flat voltage profile and smaller changes in the discharge curves at different cycles, demonstrating excellent cyclic stability after Se coating on LNO (Figure 5d,e). The coating layer reduces direct contact between the electrolyte and the active material, thus reducing the occurrence of side reactions. As shown in **Table** **1**, the electrochemical performance of Se–LNO was compared with that of reported LiNiO_2_ in the literature. It is clear that the Se‐modified LiNiO_2_ used in this study exhibits superior electrochemical performance and is competitive.

**Table 1:** Electrochemical performance of different modifications for LiNiO_2_

Modification strategy	Voltage [V]	Capacity retention [%]	Capacity decay rate cycle^−1^ [%]	References
Al doped	2.8‐4.5	81 (400th, 0.5 C)	0.048	[21]
LiCoO_2_@Co_3_O_4_ coating	3.0‐4.2	87.4 (50th, 1 C)	0.252	[51]
Graphene coating	2.8‐4.5	56.3 (500th, 1 C)	0.087	[52]
Sb doped	2.8‐4.4	86.6 (60th, 0.2 C)	0.223	[53]
Zr doped	2.7‐4.3	81 (100th, 0.5 C)	0.190	[20]
Mg/Ti doped	2.5‐4.4	77 (300th, 1 C)	0.077	[54]
Mn doped	3.0‐4.3	85.4 (200th, 0.5 C)	0.073	[55]
Se coating	2.8‐4.3	82.09 (300th, 1 C)	0.060	This work

**Figure 6:**
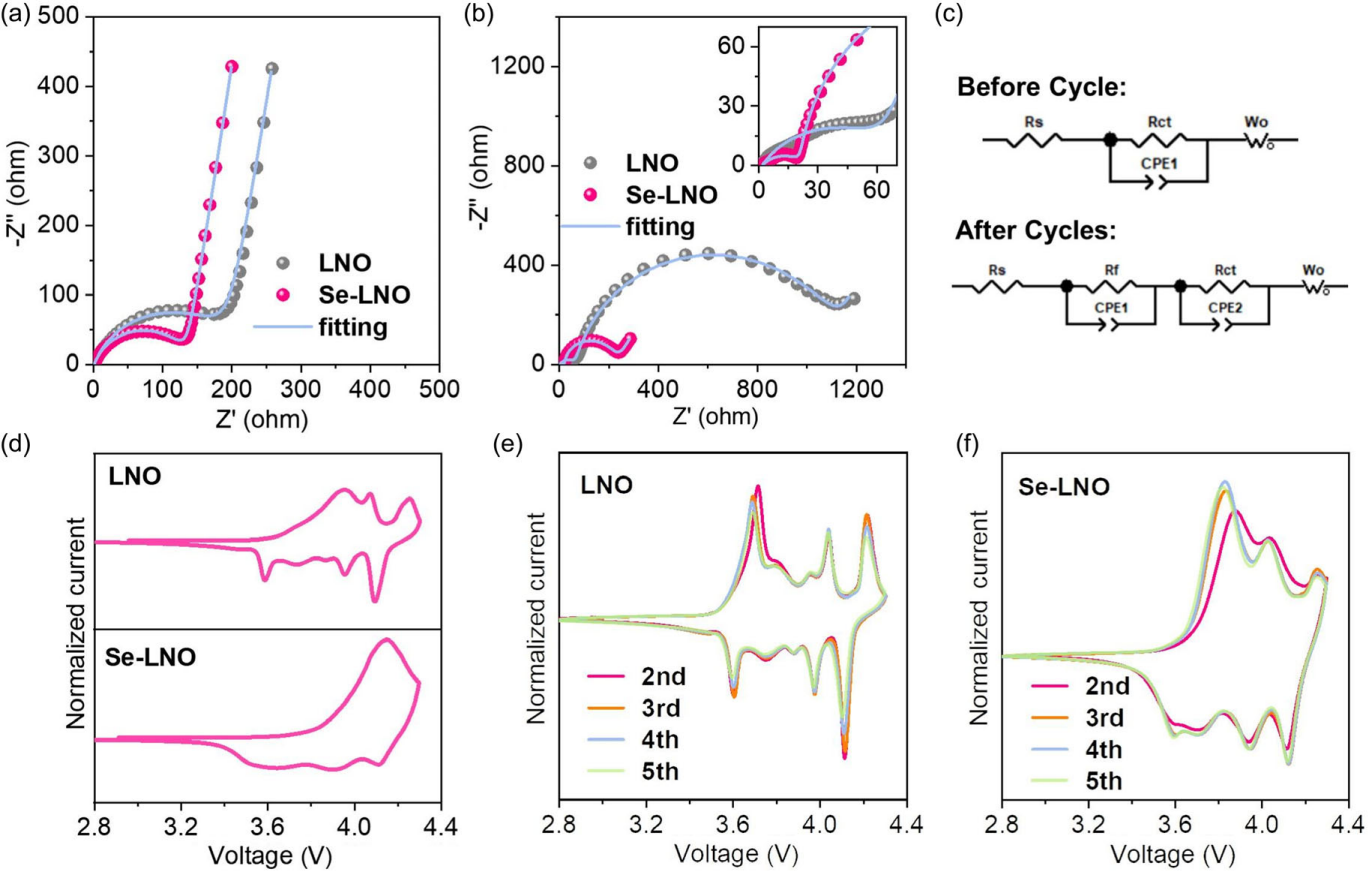
Comparison of the kinetic behavior of cathode materials before and after coating. a,b) Nyquist plots of LNO and Se‐LNO cathodes before (a) and after (b) 50 cycles and c) equivalent circuit diagram used for impedance fitting of the cell before and after cycling. d) Initial CV curve for LNO and Se‐LNO, and e,f) second to fifth cycles of LNO (e) and Se–LNO (f) at a scan rate of 0.1 mV s^−1^.

Electrochemical impedance spectroscopy (EIS) and cyclic voltammetry (CV) were used to reveal the influence of the Se coating layer on the kinetic behavior of the cathode material during the charge and discharge processes. **Figure** **6**a,b shows the Nyquist diagrams of a coin battery containing the LNO and Se–LNO electrodes before and after 50 cycles at 1 C, respectively. According to the equivalent circuit diagram in Figure 6c, the intercept on the real axis (*Z′*) represents the solution resistance (*R*_s_), the semicircle in the high‐frequency region represents the solid‐electrolyte film impedance (*R*_f_), the semicircle in the middle‐frequency region represents the charge transfer impedance (*R*_ct_), and the slope of the low‐frequency region is related to the Warburg impedance representing bulk‐phase Li^+^ diffusion.^[^ ^47^, ^48^ ^]^ The results of the fit EIS measurements are listed in **Table** **2**. Before cycling, the *R*_ct_ value of Se–LNO (133.6 Ω) was lower than that of LNO (193.7 Ω). After 50 cycles, the *R*_f_ and *R*_ct_ values of Se–LNO were 20.8 and 174.3 Ω, respectively. The *R*_ct_ of LNO was 1089.0 Ω after cycling. Se–LNO showed lower impedance values than LNO before and after cycling, indicating that the Se coating layer reduced the impedance and enhanced the charge transfer rate. The increased resistance of the LNO electrode was caused by the exacerbation of surface degradation, such as transition metal dissolution, destruction of the solid‐electrolyte interphase coatings, and the formation of disordered phase structures. However, after Se coating, surface degradation was effectively alleviated. The CV curves of the first cycle of LNO and Se–LNO in the voltage range of 2.8–4.3 V at a scanning rate of 0.1 mV s^−1^ are shown in Figure 6d. Evidently, LNO exhibited more redox peaks, indicating that it undergoes more phase changes. In contrast, Se–LNO exhibited fewer redox peaks, indicating that the Se coating layer could suppress the phase change of the cathode material during charging and discharging. The second to fifth CV curves exhibited the same effect (Figure 6e,f), which further evidences the role of the Se coating in suppressing the phase transition.

**Table 2:** EIS fitting results at charged state before and after 50 cycles at 1 C of LNO and Se‐LNO electrodes

Samples	Before cycling		After cycling		
	*R*_s_ [Ω]	*R*_ct_ [Ω]	*R*_s_ [Ω]	*R*_f_ [Ω]	*R*_ct_ [Ω]
LNO	1.2	193.7	1.7	66.0	1089.0
Se–LNO	1.3	133.6	1.8	20.8	174.3

**Figure 7:**
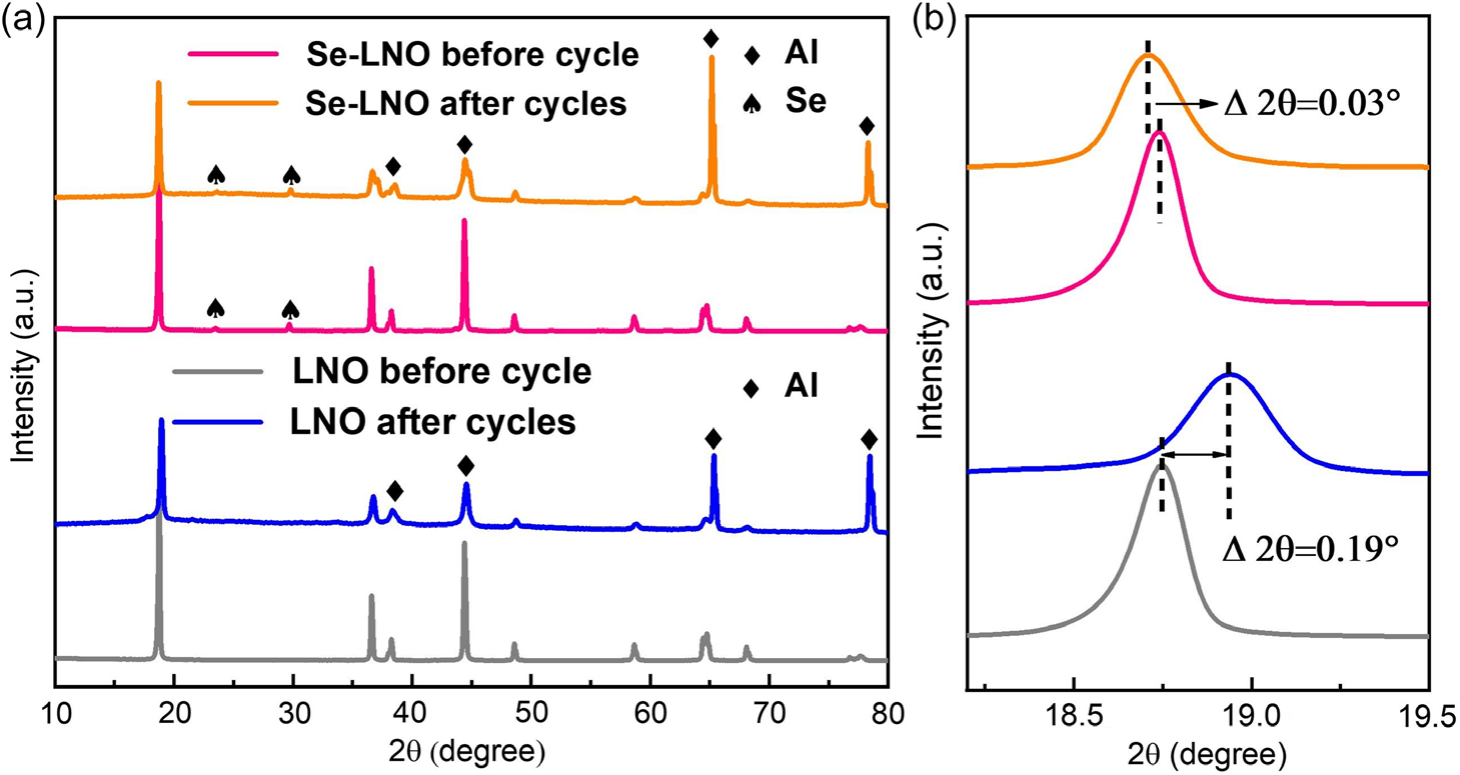
a) XRD patterns of pristine LNO and Se‐LNO after 300 cycles and b) the magnified sections of (003).

The cycled electrodes were analyzed to further investigate the influence of the Se coating on the stability of the cathode materials. **Figure** **7** shows the XRD patterns of LNO and Se–LNO before and after 300 cycles at 1 C. The Se–LNO sample exhibited the characteristic peak of Se even after cycling (Figure 7a), which indicates that the Se coating layer was not destroyed and can firmly protect the cathode materials. However, as shown in Figure 7b, the (003) peak of the LNO sample shifted to the right with an offset angle of 0.19°, which is considerably greater than that of the Se–LNO sample (offset of 0.03°), indicating that Se as a coating layer can stabilize the material structure and effectively improve the cycling performance.

**Figure 8:**
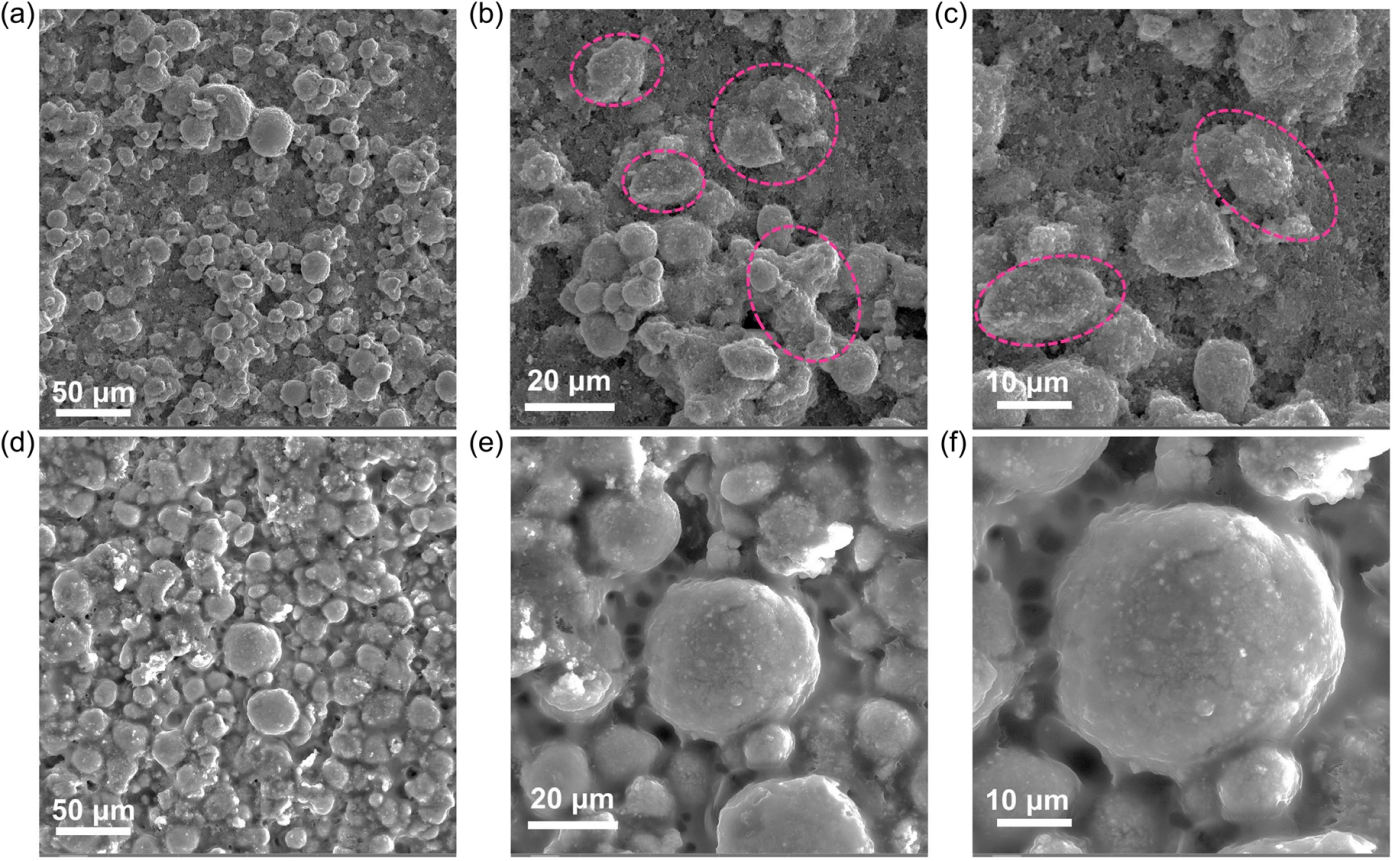
a–f) SEM images of LNO (a–c) and Se‐LNO (d–f ) after 300 cycles.

SEM images of the LNO electrode after 300 cycles are shown in **Figure** **8**a–c. Many microspheres underwent uneven fragmentation, as shown in the figure. In comparison, the Se–LNO electrode was hardly damaged after cycling, and the cathode microspheres remained intact with no visible fractures (Figure 8d–f). Direct contact with the electrolyte degrades pure LiNiO_2_ cathode materials, resulting in the dissolution of the transition metal and eventual degradation of the material microstructure.^[^ ^49^, ^50^ ^]^ Furthermore, mechanical stress induced by the change in the unit cell volume with repeated cycling causes the microspheres to fragment.^[^ ^19^, ^20^ ^]^ The Se coating layer insulates the electrolyte, protecting the cathode materials from electrolyte corrosion and thereby reducing interfacial side reactions. It also reduces mechanical stress and decreases fracture formation. Finally, it provides remarkable structural stability to Co‐free LiNiO_2_ cathode materials.

## 3 Conclusions

Se–LNO cathode materials were prepared using a simple melt‐diffusion process at low temperatures. Because of the low melting point of selenium, this coating process requires less energy and has the potential for large‐scale use. The Se coating layer minimizes the occurrence of interfacial side reactions, inhibits phase transitions, and limits particle pulverization, thereby strengthening the interfacial and internal structural stability of the cathode materials. As a result, the Se–LNO cathode material showed significantly enhanced cycling performance, with a capacity retention of 82.09% at a current density of 1 C after 300 cycles. Additionally, the modified LiNiO_2_ cathode material exhibited a lower impedance and faster charge transfer rate, indicating improved kinetic behavior. Consequently, the modified LiNiO_2_ cathode exhibited a better rate performance with specific capacities of 168.7 and 149.6 mAh g^−1^ at current densities of 2 and 5 C, respectively. Notably, the solid‐phase low‐temperature Se coating strategy reported in this study is applicable to other cathode materials and can improve their structural stability and electrochemical performance.

## 4 Experimental Section

4.1

4.1.1

##### Materials Preparation

Co‐free LiNiO_2_ cathode material was prepared by mixing spherical Ni(OH)_2_ (Zhichuan Group Co., Ltd., China) and LiOH·H_2_O precursor powders in a 1:1.05 mol mol^−1^ ratio. The mixture was precalcined at 480 °C in an oxygen environment for 6 h and then calcined at 650 °C for 12 h. Se–LNO was synthesized using a combination of solid‐phase mixing and low‐temperature sintering. First, selenium powder (99.9%, Shanghai Macklin Biochemical Co., Ltd., China) with a mass ratio of 3% with respect to LiNiO_2_ was mixed with the LiNiO_2_ powder and ground evenly using an agate mortar. Se–LNO was obtained by sintering the above‐powdered mixture at 280 °C under nitrogen for 10 min.

##### Characterizations

A D/max 2550 VB/PC X‐ray diffractometer (Rigaku, Japan) was used to analyze the crystal structures of the samples. A Gemini 500 field‐emission scanning electron microscope (Hitachi, Japan) and JEOL‐2100 F transmission electron microscope (Nippon Electron) were used to characterize the surface morphology of the samples. The powder samples were pressed into flakes and etched to different depths using Ar plasma with an Al‐Kα X‐ray radiation source, energy of 2 keV, a test spot area of 400 μm, an etched area of 2.5 μm × 2.5 μm, a single etching time of 100 s, and three etches. Subsequently, the samples were analyzed using an AXIS Ultra DLD spectrometer to determine their chemical compositions and valence states. All the XPS profiles were charge referenced to the adventitious C 1s C–C peak at 284.8 eV.

##### Electrochemical Measurements

The cathode material, conductive agent (acetylene black), and binder polyvinylidene fluoride (PVDF) were combined in an 8:1:1 mass ratio in a mortar, and the mixture was crushed to a smooth slurry with the addition of *N*‐methyl‐2‐pyrrolidone (NMP, anhydrous, 99.5%, Sigma‐Aldrich). The slurry was uniformly coated onto a clean aluminum foil and dried in a vacuum oven at 80 °C for 12 h. The aluminum foil with the material was then sliced into discs with a diameter of 12 mm and placed aside while the cathode active material load was maintained at ≈2 mg cm^−2^. A self‐prepared electrode sheet was used as the cathode, and a lithium foil with a diameter of 16 mm was used as the counter electrode. A Celgard 2500 polypropylene film was used as a separator. For the electrolyte, 1 m LiPF_6_ was dissolved in a 1:1 mass ratio of ethylene carbonate (EC) and ethyl methyl carbonate (EMC), with the addition of 2 wt% vinylene carbonate (VC). The positive electrode was a self‐prepared electrode sheet, and the counter electrode was a lithium foil with a diameter of 16 mm. The CR2032 coin cells were assembled in a glovebox filled with argon. The LAND battery test system was used to test the constant current charge and discharge of the battery in the voltage range of 2.8–4.3 V. EIS measurements and CV tests were performed on a Reference 600+ electrochemical workstation (Gamry Instruments, USA). A sine‐modulated AC potential of 5 mV was supplied to the half cell in the frequency range from 1 MHz to 1 mHz for the EIS experiments. CV testing was performed at a cutoff voltage of 2.8–4.3 V and a sweep rate of 0.1 mV s^−1^.

## Conflict of Interest

The authors declare no conflict of interest.

## Author Contributions

Y.C. and J.Z. contributed equally to this work. Y.C. took care of conceptualization, investigation, data curation, formal analysis, methodology, writing the original draft, and writing the review and editing. J.Z. conducted formal analysis, software, writing the original draft, and writing the review and editing. W.L. took care of software and writing the review and editing. F.C. took care of formal analysis and writing the review and editing. J.L. took care of writing the review and editing. F.W. took care of supervision, conceptualization, methodology, writing the original draft, and writing the review and editing.

## Data Availability

The data that support the findings of this study are available from the corresponding author upon reasonable request.
